# Getting pregnant with congenital adrenal hyperplasia: Assisted reproduction and pregnancy complications. A systematic review and meta-analysis

**DOI:** 10.3389/fendo.2022.982953

**Published:** 2022-08-31

**Authors:** Xiaoyan Guo, Yu Zhang, Yiqi Yu, Ling Zhang, Kamran Ullah, Mengxia Ji, Bihui Jin, Jing Shu

**Affiliations:** ^1^ Center for Reproductive Medicine, Department of Reproductive Endocrinology, Zhejiang Provincial People’s Hospital, Affiliated People’s Hospital, Hangzhou Medical College, Hangzhou, China; ^2^ School of Nursing, Hangzhou Medical College, Hangzhou, China; ^3^ Department of Biology, The University of Haripur, Haripur, Pakistan

**Keywords:** congenital adrenal hyperplasia (CAH), assisted reproduction technology (ART), pregnancy complication, meta-analysis, systematic review, miscarriage, abortion (induced), glucocorticoid therapy

## Abstract

Many patients with congenital adrenal hyperplasia (CAH) refrain from seeking pregnancy, suffer from infertility or worry about pregnancy complications, mainly due to genitalia abnormalities, anovulation, unreceptive endometrium and metabolic disturbances. Despite those challenges, many live births have been reported. In this systematic review, we focused on the key to successful assisted reproduction strategies and the potential pregnancy complications. We did a systematic literature search of Pubmed, Medline and Scopus for articles reporting successful pregnancies in CAH other than 21-hydroxylase deficiency, and found 25 studies reporting 39 pregnancies covering deficiency in steroidogenic acute regulatory protein, 17α-hydroxylase/17,20-lyase, 11β-hydroxylase, P450 oxidoreductase, cytochrome b5 and 3β-hydroxysteroid dehydrogenase. We summarized various clinical manifestations and tailored reproduction strategy for each subtype. Furthermore, a meta-analysis was performed to evaluate the pregnancy complications of CAH patients. A total of 19 cross-sectional or cohort studies involving 1311 pregnancies of classic and non-classic CAH patients were included. Surprisingly, as high as 5.5% (95% CI 2.3%-9.7%) of pregnancies were electively aborted, and the risk was significantly higher in those studies with a larger proportion of classic CAH than those with only non-classical patients (8.43% (4.1%-13.81%) VS 3.75%(1.2%-7.49%)), which called for better family planning. Pooled incidence of miscarriage was 18.2% (13.4%-23.4%) with a relative risk (RR) of 1.86 (1.27-2.72) compared to control. The miscarriage rate in non-classical CAH patients was not significantly different with or without glucocorticoid treatment from retrospective studies. CAH patients were also more susceptible to gestational diabetes mellitus, with a prevalence of 7.3% (2.4%-14.1%) and a RR 2.57 (1.29-5.12). However, risks of preeclampsia, preterm birth and small for gestational age were not significantly different. 67.8% (50.8%-86.9%) CAH patients underwent Cesarean delivery, 3.86 (1.66-8.97) times the risk of the control group. These results showed that fertility is possible for CAH patients but special care was necessary when planning, seeking and during pregnancy.

**Systematic Review Registration:**

PROSPERO https://www.crd.york.ac.uk/PROSPERO/display_record.php?RecordID=342642, CRD42022342642.

## 1 Introduction

Congenital adrenal hyperplasia (CAH) is a family of autosomal recessive diseases caused by defects of enzymes in adrenal steroidogenesis, which is the most frequent monogenic disorder affecting sexual development and fertility (1). The different types and remaining activity of the mutant enzyme lead to a spectrum of clinical presentations, including the salt-wasting form (SW), the simple virilizing form (SV), and the non-classical form (NC). CAH may affect both male and female fertility. The main cause of male infertility in CAH is testicular adrenal rest tumors (TARTs) (2, 3), which are benign, bilateral tumors in rete testis developed under the trophic effects of chronic adrenocorticotropic hormone (ACTH) elevation, compressing the seminiferous tubules (4). In our study, we focused on fertility in female CAH patients. A series of obstacles lie in patients’ attempts at pregnancy. Classical CAH women might have malformations of external genital organs such as labial fusion and clitoral hypertrophy, which render sexual intercourse unpleasant or prohibitive even after corrective surgery. While non-classical patients might be asymptomatic during childhood, persistently elevated progestogens could lead to anovulation, unreceptive endometrium, and unfavorable cervical mucus, resulting in irregular menses and infertility (5).

Since the first live birth was achieved by a patient with 21-hydroxylase deficiency (21OHD) in 1956 (6), the fertility rate has greatly improved over the past 60 years. In the 1980s, only half of 80 classical 21OHD women reported adequate vaginal introitus to be heterosexually active, among whom 15 gave birth (7). However, in the 2020s, the fertility rates of simple virilizing (41.8%) and non-classical (40.8%) patients were greatly improved to be comparable to those of the common population (45.8%) in Sweden, although only 8.1% of salt-wasting patients had biological children (8). Fertility treatment for 21OHD patients has been summarized (5). For patients with rarer types of CAH other than 21OHD, recent advances in genetics and assisted reproductive technology (ART) aided their diagnosis and fertility, who presented with a drastically different clinical picture and required tailored fertility treatment.

Although many pregnancies went uneventful, clinicians and patients worried about the risk of pregnancy complications due to significantly higher incidence of obesity, hypertension, and insulin resistance before pregnancy and corticoid supplementation during pregnancy (9). Several studies have reported an increased risk of gestational diabetes mellitus (GDM), small for gestational age (SGA) (10), and cesarean delivery (8), while others report uneventful pregnancies. Also, some studies have recommended glucocorticoid use in the non-classical type of CAH to lower the miscarriage rate (11, 12), but the study by Eyal et al. (13) suggested that glucocorticoid treatment made no difference.

In this systematic review and meta-analysis, we aimed to summarize the ART use in female patients with rare types of CAH based on case reports of successful live births. Furthermore, we performed a series of meta-analyses to evaluate the prevalence of pregnancy complications in CAH, including miscarriage, elective abortion, GDM, preeclampsia, preterm birth, SGA, and cesarean delivery. We then calculated the relative risk of pregnancy complications in CAH patients compared to the general population. We further compared the effect of glucocorticoid treatment on preventing miscarriage in the non-classical type of CAH.

## 2 Methods

### 2.1 Search strategy and selection criteria

**Figure 1:**
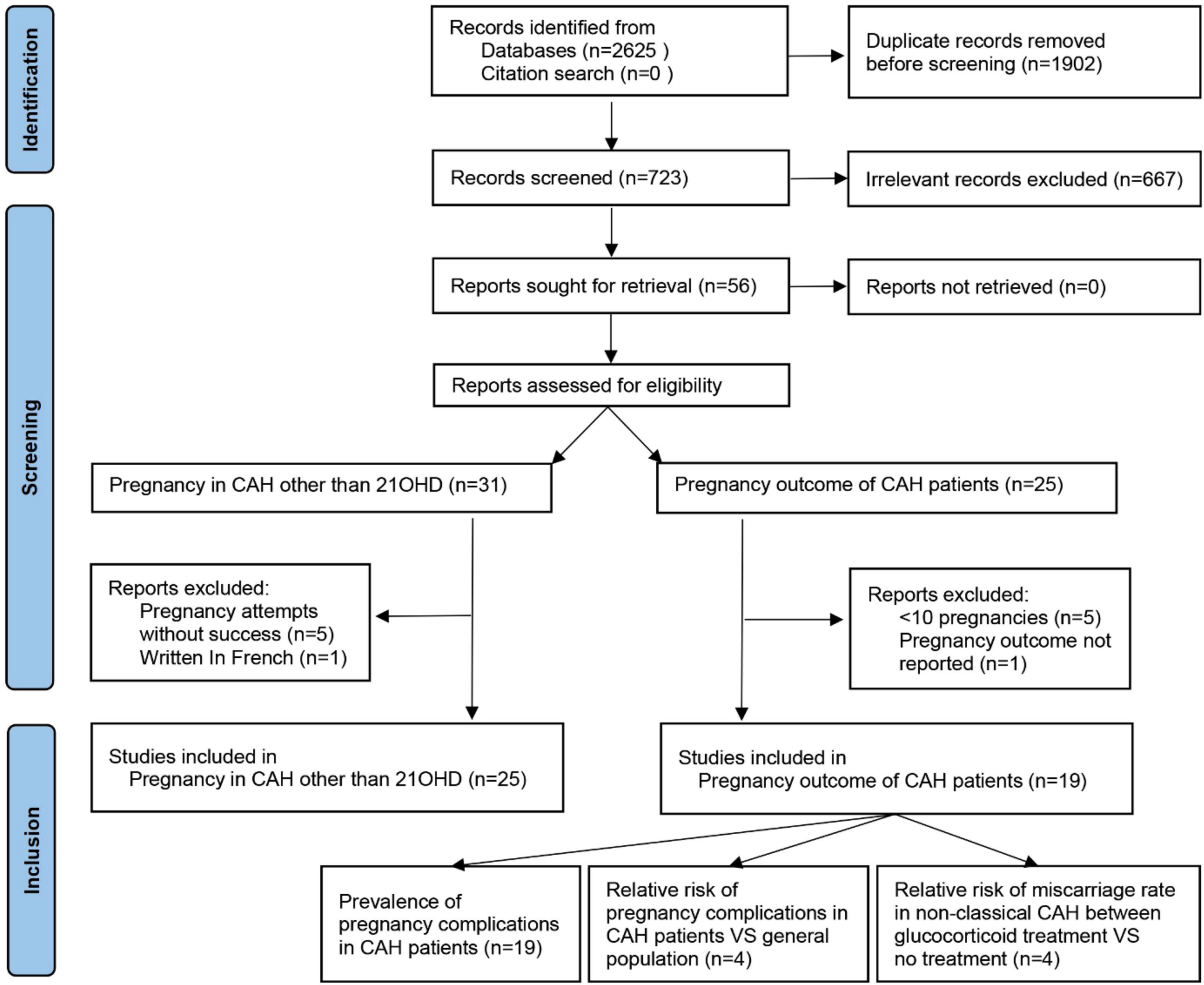
PRISMA flowchart of literature search and selection. PRISMA, Preferred Reporting Items for Systematic Reviews and Meta-Analyses.

PubMed, Medline, Scopus, and forward and backward citations were searched to identify studies between database inception and 1 June 2022. Search terms are listed in **Appendix 1**, and the language was restricted to English. A total of 723 titles and abstracts were screened after the removal of duplicates (**Figure 1**). For pregnancies in CAH other than 21OHD, the inclusion criteria were case reports with clinical pregnancy achieved, and four studies reporting attempts without success (14–18) and one written in French (19) were excluded. For pregnancy complications in CAH, the inclusion criteria were cross-sectional, case–control, or cohort in design with pregnancy outcomes reported (n = 21). One paper without detailed pregnancy outcomes (20) and five case series with less than 10 pregnancies (21–25) were excluded for fear that sampling bias would be dramatic considering the occurrence of common complications. This study followed Preferred Reporting Items for Systematic Reviews and Meta-Analyses (PRISMA) guidelines (26) with a checklist in **Appendix 2** and was registered on https://www.crd.york.ac.uk/PROSPERO/display_record.php?RecordID=342642 PROSPERO (CRD42022342642).

### 2.2 Quality assessment and data extraction

The criteria for risk of bias assessment were adapted from the Hoy tool (27), with a maximum score of 8 for prevalence studies and a maximum score of 10 for prevalence and risk studies. Quality assessment (**Appendix 3**) and data extraction were independently done by two reviewers and cross-checked. Discrepancies were resolved through discussion with the other authors. For studies reporting pregnancy complications, the primary outcomes of interest were miscarriage, elective abortion, GDM, preeclampsia, preterm birth, SGA, and C-section. When calculating the rate of miscarriage and elective abortion, the denominator was total clinical pregnancies. However, the rates of other complications were calculated within ongoing pregnancies. Incomplete follow-up of the patients was excluded from the relevant analyses.

### 2.3 Data analysis

Given that there are 0% and 100% in the prevalence of pregnancy complications, prevalence rates were calculated from raw proportions after the Freeman–Tukey double-arcsine transformation (28), and the Shapiro test showed normal distribution. The inverse variance method was used for pooling based on a random-effects model (29). To measure dichotomous outcomes, a relative risk (RR) and 95% confidence interval (CI) were calculated using the Mantel-Haenszel method based on a random–effects model. If there was a cell count of zero, 0.5 is added to each cell frequency to correct for continuity. I^2^ was used to estimate heterogeneity, and an I^2^ value > 50% indicated significant heterogeneity. Subgroup analyses were performed according to non-classical or assorted types of patients. Egger’s test was used to assess potential publication bias whenever the number of studies was sufficient, with p < 0.1 indicating significance. Analyses and forest plots were done with R (4.2.0).

## 3 Results

### 3.1 Assisted reproductive technology for various types of congenital adrenal hyperplasia

**Figure 2:**
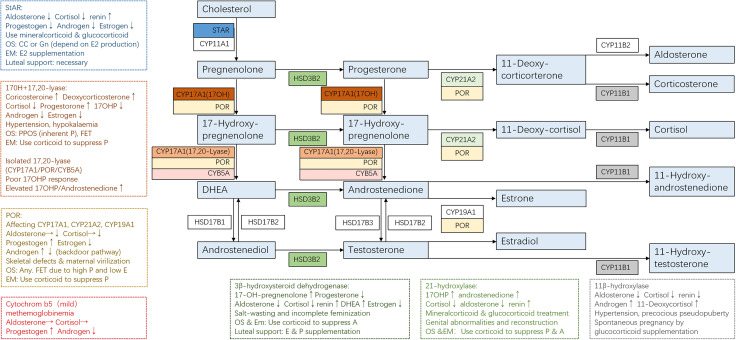
Adrenal steroidogenesis and ART use in different CAH subtypes. Clinical manifestations and corresponding protocols are summarized from case reports of pregnancies and are listed in dashed borders, but the actual situation varies from person to person. OS, ovarian stimulation; CC, clomiphene; Gn, gonadotropin; PPOS, progestin-primed ovarian stimulation; EM, endometrium preparation; FET, frozen embryo transfer.

The clinical manifestation of CAH might vary significantly depending on the mutations and adherence to treatment. For some non-classical patients, spontaneous pregnancy could be achieved simply by optimizing glucocorticoid and mineralocorticoid therapy (13). On the contrary, classical patients usually present a tricky situation where genetic diagnosis helped to give us a clear understanding of their underlying pathophysiology and was essential to developing an appropriate therapeutic strategy for better follicular, endometrium, and corpus luteum development (**Figure 2** and **Table 1**).

**Table 1:** Pregnancies in CAH other than 21-hydroxylase deficiency.

Gene	Author	Year	Mutation	Type	External genitalia	Menses	Other	Ovarian stimulation	Trigger	Embryo transfer	Corticoid	Endometrial preparation	Luteal phase	Pregnancy complications
StAR	Khoury	2009	Homo p.L275P	C	Female	Regular, without ovulation	/	CC	No	/	No	No	No	Miscarriage at 6 weeks
							/	CC	No	/	No	No	P	Quadruple pregnancy with 1 naturally lost at 7 weeks and 1 feticide at 8 weeks; gestational hypertension; preterm birth
							/	CC	No	/	No	No	P	Preterm birth
	Sertedaki	2009	Homo p.K236delfs*43	C	Female	Irregular	Ovarian cyst	Luteal phase GnRH-a	hCG	Fresh	PDS	E2	E2+P	Husband -/K236delfs*43, chorionic villus biopsy after PGT, C-section
	Albarel	2016	Homo p.T240Sfs*81	C	Female	Amenorrhea	Obese	GnRH-a	hCG	FET	HCT+FC	E2	E2+P	Miscarriage at 8 weeks
								/	/	FET	HCT+FC	E2	E2+P	No
	Hatabu	2019	p.Q258*/p.R272C	NC	Female	Regular	/	No	No	/	No	No	No	C-section
							/	No	No	/	No	No	No	C-section
			p.Q258*/p.M225T	C	Female	Irregular	Ovarian cyst	CC	No	/	NR	No	No	C-section
CYP17A1 (17OH+ 17, 20-lyase)	Ben-Nun	1995	NR	C	Infantile	Amenorrhea	BP↑, K↓	Donated oocyte	/	FET	DXM	Estradiol implants	E2+P	Twin pregnancy, HELLP syndrome, C-section, preterm birth, only one survived
	Levran	2003	NR	NC	Female	Irregular	/	GnRHa	hCG	FET	DXM	GnRHa-HRT	E2+P	Triplet live birth
	Bianchi	2016	p.W406R/p.P428L	NC	Female	Amenorrhea	BP↑	GnRHa	hCG	FET	DXM	GnRHa-HRT	E2+P	C-section at 30 weeks due to acute fetal distress and a true umbilical knot
	Yang	2017	Homo p.V236G	C	Infantile	Amenorrhea	BP↑, ovarian cyst	Inherent P+HMG	hCG	FET	DXM	E2	E2+P	HELLP syndrome, C-section, preterm birth
	Falhammar	2018	Homo exon 1-6 deletion	C	Female	Amenorrhea	BP↑, K↓, obese	NR	NR	NR	PDS	NR	NR	C-section
	Kitajima	2018	Homo p.S54del	NC	Female	Amenorrhea	BP↑, ovarian cyst	GnRHa	hCG	FET	DXM	E2	E2+P	No
										FET	DXM	E2	E2+P	Massive intrapartum hemorrhage due to placenta accreta
	Xu	2022	Homo p. R496C	NC	Female	Amenorrhea	BP↑	PPOS	hCG	FET	DXM	GnRHa-HRT	E2+P	No
			p. I332T/p. D487_F489del	NC	Female	Irregular	Ovarian cyst	inherent P+CC+HMG	hCG+ GnRH-a	FET	DXM	E2	E2+P	Cleft lip and palate, termination
CYP17A1 (17, 20-lyase)	Blumenfeld	2021	Homo p.E305G	NC	Female	Irregular	Ovarian cyst	GnRHa	hCG	FET	PRED	GnRHa-HRT	E2+P	Miscarriage
								/	/	FET	PRED	GnRHa-HRT	E2+P	No
POR	Song	2018	Homo p.Y326D	NC	Female, vaginal atrasia	Irregular	Unicornuate uterus, ovarian cyst	LE+HMG	NR	FET	DXM	E2	E2+P	No
	Papadakis	2020	c.1249-1G>C/c.1324C>T	NC	Female	Irregular	Ovarian cyst	GnRH-a	hCG	FET	HCT	NR	E2+P	Twin pregnancy
			p.Gln609*/p.W620S	NC	Female	Irregular	Ovarian cyst	GnRH-antagonist	hCG	FET	DXM	NR	E2+P	Preeclampsia, C-section
	Zhang	2020	IVS14-1G>C/p.V603_Q606del	NC	Aberrant	Irregular	Ovarian cyst	luteal phase GnRH-a	hCG	FET	PDS	E2	E2+P	Twin pregnancy, chronic hypertension in pregnancy, preterm, C-section
	Pan	2021	p.R457H/p.P399_E401del	NC	Female	Amenorrhea	Ovarian cyst, mild skeletal malformation	GnRH-a	hCG	FET	DXM	E2	E2+P	Twin pregnancy, C-section
CYB5A	Leung	2020	Homo Y35*	NC	Female	Regular	Methemoglobinemia	No	No	/	No	No	No	No
HSD3B2	Rojansky	1991	NR	NC	Female	Irregular	Hirsutism, obese	HMG	hCG	FET	DXM	CC	NR	No
CYP11B1	Toaff	1975	NR	NC	Female	Regular	/	No	No	/	No	No	No	No
								No	No	/	DXM	No	No	No
	Simm	2007	DS+2/p.G444D	C	Aberrant	Irregular	Insulin resistance	CC	NR	/	DXM	NR	NR	Pregnancy-induced hypertension
	Parajes	2010	Homo p.P159L	NC	Female	Regular	Hirsutism	No	No	/	PDS	No	No	4 uncomplicated pregnancies
	Menabo	2014	p.R143W/p.A306V	NC	Female	NR	Hirsutism	No	No	/	PDS	No	No	NR
	Mooij	2015	Homo p.R143W	NC	Female	Irregular	Hirsutism	NR	NR	/	PDS	NR	NR	Twin pregnancy, miscarriage at 17 weeks
								NR	NR	/	PDS	NR	NR	4 uncomplicated pregnancies
	Zacharieva	2019	p.D480Tfs*2/p.V316M	NC	Female	Regular	Hirsutism, BP↑	No	No	/	DXM	No	No	Chronic hypertension, C-section
								No	No	/	DXM	No	No	Elective abortion
	Krishnan	2021	NR	C	Aberrant	Regular	Hirsutism, BP↑	No	No	/	PDS	No	No	Preeclampsia, C-section, preterm birth

NC, non-classical; C, classical; NR, not reported; CC, clomiphene, LE, letrozole; HCT, hydrocortisone; PDS, prednisolone; DXM, dexamethasone; FC, fludrocortisone.

*, nonsense mutation.

#### 3.1.1 Steroidogenic acute regulatory protein

Steroidogenic acute regulatory protein (StAR) accounts for about 86% of the transfer of cholesterol from the outer to inner mitochondrial membrane, where it is converted to pregnenolone after the cleavage of the side chain by P450 side-chain cleavage enzyme (P450scc, encoded by gene *CYP11A1*) (30). This is the initial and rate-limiting step in steroidogenesis. Therefore, mutated StAR impedes steroidogenesis and accumulates cholesterol, causing lipoid CAH, which is the most severe form of steroidogenesis and is characterized by the near absence of all steroids, high basal ACTH, and plasma renin activity, and grossly enlarged adrenals stacked with cholesterol and cholesterol esters (31).

StAR is expressed in the gonads and adrenal glands but not in the placenta, so affected 46,XX individuals will manifest at birth with glucocorticoid and mineralocorticoid deficiency and puberty sex steroid production problems. Although the steroidogenic pathway is affected, germ cell migration and maturation are theoretically normal. According to the reported cases (32–35), female lipoid CAH patients with glucocorticoid and mineralocorticoid substitution enter puberty normally because a low level of estrogen produced by the StAR-independent pathway is enough to support secondary sex characters and menarche. However, the higher demands for estrogen necessary for early follicular development, the positive feedback of luteinizing hormone (LH) surge, and endometrium growth cannot be met. Anovulation, high LH/follicle-stimulating hormone (LH/FSH) ratio, and ovarian cysts may resemble polycystic ovary syndrome (PCOS), but low testosterone should raise attention. Ovulation induction is necessary when the patient has irregular menses. A human chorionic gonadotropin (hCG) stimulation or a clomiphene test might be performed to see the capacity for estrogen production, with spontaneous puberty and regular menses being signs of responsiveness. If the patient is responsive, clomiphene might be used to induce ovulation. If the patient failed to produce enough estradiol for endometrium proliferation after clomiphene (CC), extra estrogen administration is beneficial, and ART is recommended. Progesterone supplementation is necessary for luteal support, and spontaneous abortion occurred in one patient without progesterone supplementation. Luteal support should be sustained until placental function takes over.

Unlike StAR, P450scc is present in all steroidogenic tissues including the placenta. Considering that progesterone produced by the placenta was necessary for preventing miscarriage since the second trimester, few fetuses with P450scc mutations reached term gestation. Most reported cases were in 46,XY patients with complete sex reversal with adrenal insufficiency (36). Non-classic P450scc deficiency resembles non-classic lipoid CAH in terms of hormonal presentations. However, all patients with P450scc deficiency have been reported to have normal-sized or small adrenals, in contrast to the massive adrenal enlargement in lipoid CAH. No pregnancy has been achieved in patients with P450scc deficiency.

#### 3.1.2 CYP17A1

17α-Hydroxylase (17OH) and 17,20-lyase (17,20-desmolase) are considered two separate functions of the same enzyme P450c17 encoded by gene *CYP17A1*, the function of which depends on the local factors (37, 38). Therefore, mutations in CYP17A1 could cause three different forms of enzymatic deficiency: 1) combined deficiencies of the two functions, which is the most common form, 2) isolated 17OH deficiency, and 3) isolated 17,20-lyase deficiency. Patients with double deficiency (39–45) suffer from hypertension and impaired glucocorticoid production. Low estradiol levels might lead to infantile genitalia. High levels of progesterone inhibit the GnRH/LH pulse frequency and result in amenorrhea, unreceptive endometrium, and ovarian cysts. To date, no spontaneous pregnancy has been reported in women with 17OHD. Live birth has only been achieved by ART in 17OHD due to embryo–endometrium asynchrony under high progesterone. As for ovarian stimulation, the inherently high progesterone levels render the protocol a progestin-primed one in essence. Frozen embryo transfer or the “freeze all” strategy is a great advantage in the high P situation. Corticoid administration rather than the gonadotropin-releasing hormone agonist (GnRHa) is the key to suppressing P and ensuring endometrium proliferation and the proper timing for embryo implantation.

It is noteworthy that the activities of both 17α-hydroxylase and 17,20-lyase are dependent on the availability of cytochrome P450 oxidoreductase (POR), which is the obligatory electron transfer flavoprotein. Other flavoproteins can partially substitute POR for the 17-hydroxylase activity but not the 17,20-lyase activity, so 17,20-lyase is particularly vulnerable to the abundance and function of POR (46). In addition, the optimal 17,20-lyase reaction requires the facilitation of cofactor protein cytochrome b5, which stimulates the rate of the reaction to over 10-fold (47). Therefore, isolated 17,20-lyase deficiency is a syndrome, which may be caused by specific mutations in the CYP17A1 (p.R347H, p.R347C, p.R358Q, and p.E305G), POR (p.G539R), and CYB5A (p.W27X and p.H44L) (14). Isolated 17,20-lyase deficiency is characteristic of an elevated ratio of 17OHP to androstenedione and showed low cortisol levels under the stimulation of ACTH or 17OHP. Only one pregnancy has been reported in isolated 17,20-lyase deficiency (38), demonstrating persistent progesterone and low estrogen. The patient experienced three failed *in vitro* fertilization (IVF) cycles and retrieved 37 oocytes using the long GnRHa protocol for the fourth time. Due to the high serum progesterone concentration, all embryos were cryopreserved. Hormone replacement therapy was used to prepare the endometrium due to inherently low estrogen levels, along with prednisone 30 mg/day. Live birth was achieved after two cycles of embryo transfer.

#### 3.1.3 POR

The P450 oxidoreductase (encoded by *POR* gene) transfers electrons from reduced nicotinamide adenine dinucleotide phosphate (NADPH) to all P450 enzymes, including P450c17 (17OH/17, 20-lyase), P450c21 (21-OH), and P450aro (aromatase). POR deficiency diverts steroids into the “backdoor pathway” of dihydrotestosterone biosynthesis. The extent to which various enzymes are affected depends on the specific mutations of *POR* gene, resulting in high clinical variability. Phenotypes of female patients include high levels of P (100%), pregnenolone (100%), 17OHP (96%), corticosterone (83%) and deoxycorticosterone (70%), adrenal insufficiency after ACTH stimulation (78%), skeletal malformations (84%), and ovarian cysts (39%) (48). Given that POR was expressed in the placenta, reduced activity of placental aromatase might lead to intrauterine androgen excess causing virilized genitalia in affected female individuals (78%) and maternal virilization during pregnancy (21%). Clinical manifestations might resemble both 21OHD (abnormal genitalia) and 17OHD (elevated progesterone levels and low estradiol levels). The unreceptive endometrium under high P rendered ART application and frozen embryo transfer mandatory. Ovarian stimulation protocol did not seem to affect oocyte quality despite the significantly low E2 levels. Just like 17OHD, frozen embryo transfer and corticoid supplementation to suppress P during endometrium preparation were consistently utilized by all patients (49–52).

#### 3.1.4 CYB5A

Cytochrome b5 serves as an allosteric cofactor favoring 17,20-lyase reaction. CYB5A mutation leads to an isolated and partial 17,20-lyase deficiency. An important feature in diagnosis is normal cortisol response but absent or blunted 17OHP response after ACTH stimulation. Patients manifest methemoglobinemia, with normal sexual development, regular menses, and spontaneous pregnancy (44).

#### 3.1.5 HSD3B2

Type II 3β-hydroxysteroid dehydrogenase deficiency (3βHSDIID) impaired both adrenal and gonadal steroidogenesis. Patients have excess production of androgen precursors, which are converted to active androgens in the peripheral tissues by the normal type I 3βHSD. Clinical presentation might vary, ranging from severe neonatal salt-wasting with normal external genitalia and regular menses to complete dependence on estradiol therapy to undergo complete feminization and menses (53). Only one pregnancy has been reported with HSD3B2 mutation (54). The patient had normal genitalia and signs of hirsutism and obesity. She presented with increased 17-OH-pregnenolone and DHEAS with normal electrolytes and blood pressure. With dexamethasone treatment, ovarian stimulation with HMG and hCG went smoothly, and the pregnancy was uneventful after frozen embryo transfer, resulting in the delivery of a healthy full-term female infant.

#### 3.1.6 CYP11B1

11β-hydroxylase (CYP11B1) converts 11-deoxy-cortisol to cortisol and converts androstenedione and testosterone to their 11-hydroxy forms. Therefore, 11OHD results in hyperandrogenism, glucocorticoid deficiency, and hyporeninemic hypertension due to elevated mineralocorticoid precursors. Nevertheless, the degree of hyperandrogenism did not correlate with the extent of mineralocorticoid excess. Hyperandrogenism in 11OHD may present with precocious pseudopuberty, characterized by accelerated growth during childhood and reduced final height. Androgen excess may also suppress later stages of follicular development and impair endometrial receptivity, despite that some individuals may have regular menses. Correct diagnosis of non-classical 11OHD was essential because immediate restoration of fertility and rapid normalization of the blood pressure could be achieved after the initiation of corticosteroid therapy (19, 55–61).

### 3.2 Pregnancy complications of congenital adrenal hyperplasia patients

**Figure 3:**
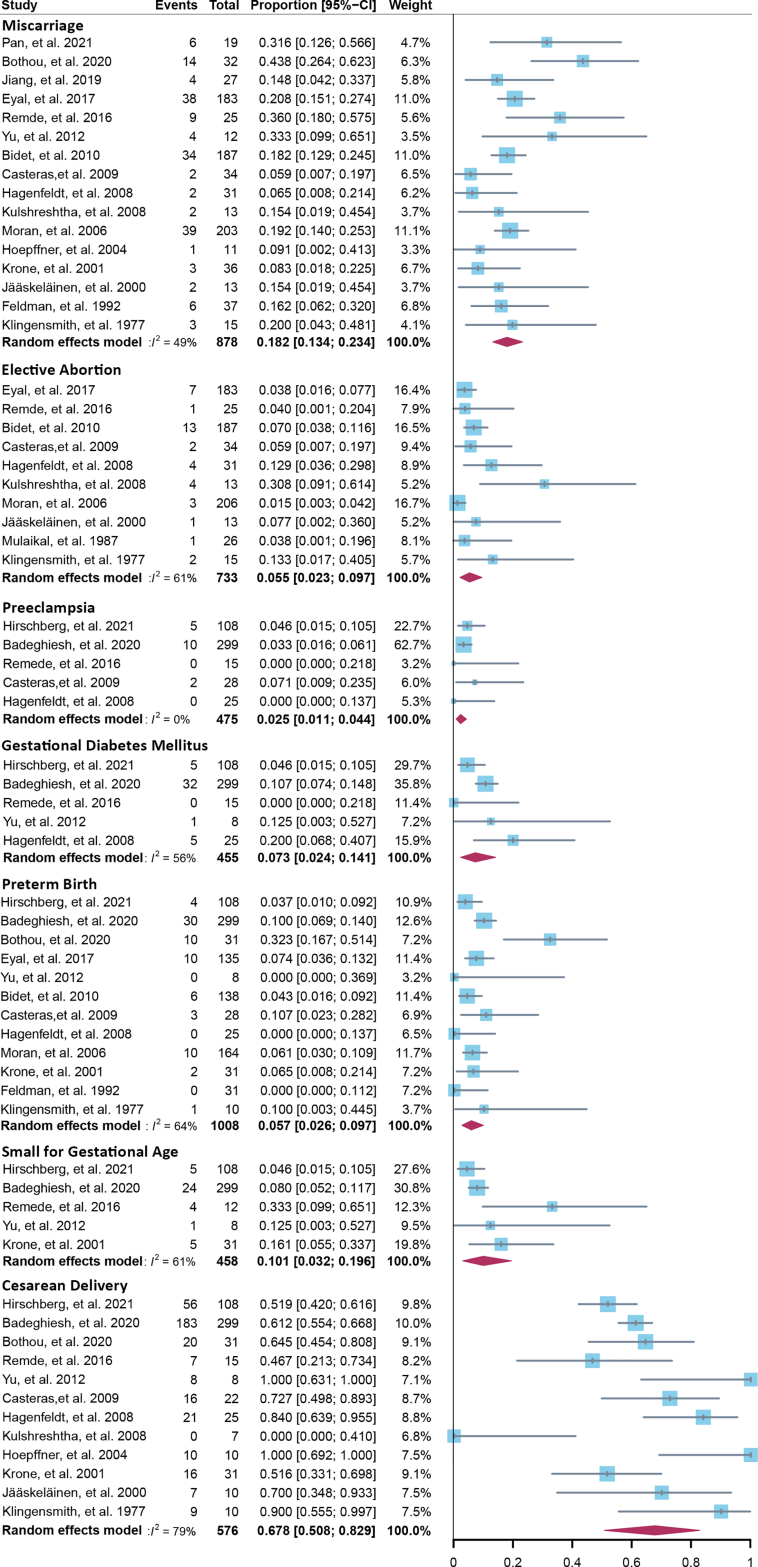
Prevalence of pregnancy complications in CAH patients. CAH, congenital adrenal hyperplasia.

**Figure 4:**
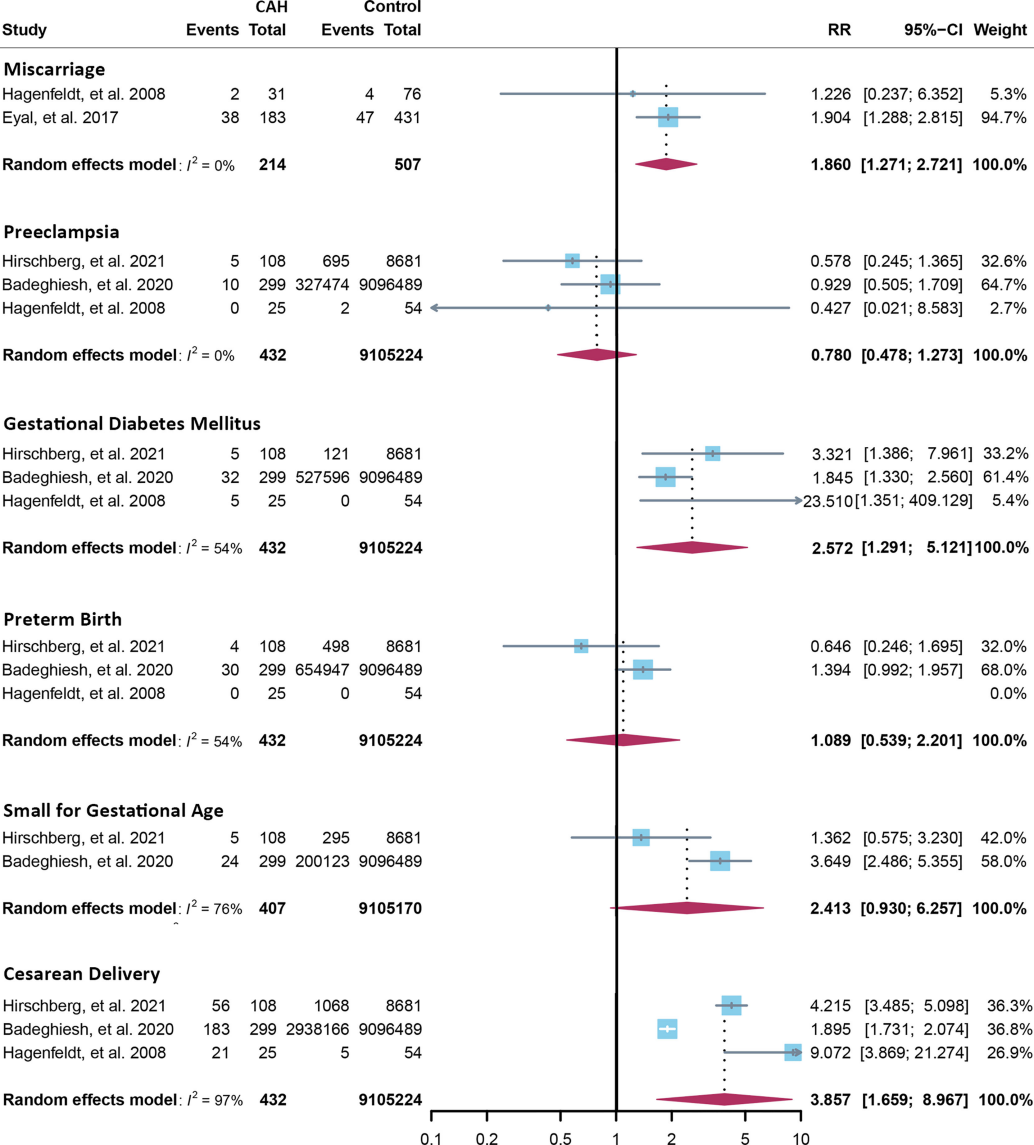
Relative risk of pregnancy complications in CAH. CAH, congenital adrenal hyperplasia.

**Figure 5:**
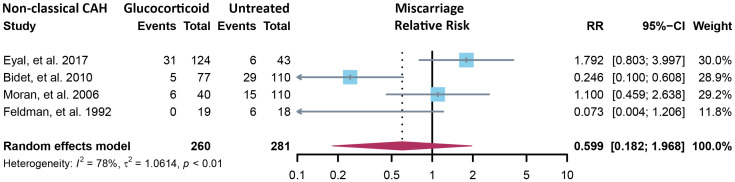
Relative risk of miscarriage in the non-classical form of CAH between glucocorticoid treatment and non-treatment. CAH, congenital adrenal hyperplasia.

Fourteen cross-sectional and five cohort studies were included (7, 8, 10–13, 62–74), reporting outcomes of 1,311 pregnancies of CAH patients. The study characteristics of included studies are listed in **Table 2**. The mean pregnancy age ranges from 23 to 31.8 years, and the mean body mass index ranges from 21.3 to 26.9 kg/m^2^. Twelve studies consisted of mixed types of CAH, while seven studies focused on the non-classical type. Prevalence and relative risk of pregnancy complications are summarized in **Figures 3** and **4**, respectively. Sixteen studies reported miscarriage rate, rendering a pooled prevalence of 18.2% (95% CI 13.4%–23.4%) with a medium heterogeneity. Subgroup analysis did not show a significant difference in miscarriage rate between non-classical type and assorted type. Two studies provided the relative risk of miscarriage compared to the general population, and the pooled relative risk was 1.86 (1.27–2.72). The risk of miscarriage in non-classical CAH patients was not significantly influenced by glucocorticoid treatment, as shown in **Figure 5**. Another major reason for early termination of pregnancy is elective abortion, accounting for as high as 5.5% (2.3%–9.7%) of CAH pregnancies. Subgroup analysis revealed that the rate of elective abortion among studies of non-classical type was significantly lower than in studies of assorted types (3.75% (1.2%–7.49%) vs 8.43% (4.1%–13.81%), p = 0.026).

**Table 2:** Characteristics of studies included in the meta-analysis.

Study	Year	Study design	Country	N of pregnant patients (CAH subtypes)	N of pregnancies (CAH subtypes)	Age (years)	BMI (kg/m^2^)	Pair of twins	Corticoid usage	Control group
Hirschberg et al.	2021	Cohort	Sweden	61 (26 SV + 8 SW + 16 NC + 11?)	108 (NR)	28.1 (4.9)	23.3 (3.5)	0	NR	Age-matched controls
Hagenfeldt et al.	2008	Cohort	Sweden	16 (9 SV + 2 SW + 3 NC + 2?)	31 (19 SV + 3 SW + 3 NC + 4?)	30	NR	0	Yes	Age-matched controls
Badeghiesh et al.	2020	Cohort	USA	NR	299 (NR)	23.1% > 35	7.7% obese	8	NR	General population
Remde et al.	2016	Cohort	Germany	12 (5 SV + 2 SW + 5 NC)	25 (6 SV + 3 SW + 16 NC)	NR	24	1	Yes	Autoimmune adrenalitis
Bothou et al.	2020	Cohort	8 countries	NR	32 (NR)	31.8 (6.1)	25.7 (4.6)	0	Yes	Addison disease or secondary adrenal insufficiency
Yu et al.	2012	Cross-sectional	China	8 (5 SV + 3 NC)	12 (6 SV + 6 NC)	31.3 (3.3)	NR	0	Yes	
Casteras et al.	2009	Cross-sectional	UK	21 (13 SV +8 SW)	34 (20 SV + 14 SW)	27.3 (5.4)	26.9 (6.1)	0	Yes	
Hoepffner et al.	2004	Cross-sectional	Germany	9 (4 SV + 5 SW)	11 (5 SV + 6 SW)	26.1 (3.3)	26.6 (3.9)	0	Yes	
Krone et al.	2001	Cross-sectional	Germany	18 (12 SV + 1 SW + 5 NC)	36 (24 SV + 3 SW + 9 NC)	27.9 (5.2)	23.7 (3.2)	0	Yes	
Jääskeläinen et al.	2000	Cross-sectional	Finland	9 (8 SV + 1 SW)	13 (12 SV + 1 SW)	30.6 (2.9)	25	0	Yes	
Mulaikal et al.	1987	Cross-sectional	USA	16 (15 SV + 1 SW)	26 (25 SV + 1 SW)	NR	NR	0	Yes	
Klingensmith et al.	1977	Cross-sectional	USA	10 (8 SV + 2 SW)	15 (13 SV + 2 SW)	26.3	NR	0	Yes	
Pan et al.	2021	Cross-sectional	China	NR	19 NC	29.9 (2.9)	22.1 (2.9)	0	Yes	
Jiang et al.	2019	Cross-sectional	China	20 NC	27 NC	30.8 (3.7)	21.3 (2.3)	0	Yes	
Kulshreshtha et al.	2008	Cross-sectional	India	5 NC	13 NC	23.0 (3.5)	NR	0	Yes	
Eyal et al.	2017	Cross-sectional	USA	72 NC	183 NC	30.7 (4.9)	24.4 (4.6)	5	Miscarriage: 6/43 of usage group vs 31/124 of non-usage group	
Bidet et al.	2010	Cross-sectional	France	85 NC	187 NC	26.7 (8.9)	24.0 (4.6)	3	Miscarriage: 5/77 of usage group vs 29/110 of non-usage group	
Moran et al.	2006	Cross-sectional	9 countries	104 NC	206 NC	29.7 (9.7)	NR	4	Miscarriage: 4/65 of usage group vs 35/138 of non-usage group	
Feldman et al.	1992	Cross-sectional	France	20 NC	37 NC	24.6 (5.2)	NR	0	Miscarriage: 0/19 of usage group vs 6/18 of non-usage group	

CAH type is categorized as the salt-wasting form (SW), the simple virilizing form (SV), and the non-classic form (NC).

?, unknown; NR, not reported.

Within ongoing pregnancies, CAH patients were more susceptible to gestational diabetes mellitus, with a prevalence of 7.3% (2.4%–14.1%) and an RR of 2.57 (1.29–5.12). However, risks of preeclampsia, preterm birth, and small gestational age were not significantly different, with a prevalence of 2.5% (1.1%–4.4%), 5.7% (2.6%–9.7%), and 10.1% (3.2%–19.6%), respectively. Alarmingly, 67.8% (50.8%–86.9%) of CAH patients underwent cesarean delivery, 3.86 (1.66–8.97) times the risk of the control group. Despite the significant heterogeneity, bias assessment showed asymmetry in the funnel plot (Egger’s test p = 0.40). Subgroup analysis according to the subtype of CAH was not appropriate for preeclampsia, GDM, SGA, and C-section with only 1 or 0 study focusing on the non-classical type, and subgroup analysis for preterm birth showed no significant difference.

## 4 Discussion

In this review, we systematically summarized the pregnancy case reports of CAH other than 21OHD and elaborated the customized fertility treatment for each subtype. To the best of our knowledge, there has been no such systematic review despite several reviews in this aspect (5, 53, 75, 76). In addition, we calculated the pooled prevalence and relative risk of pregnancy complications in CAH patients for the first time, which responded to the worries of CAH patients and controversies of researchers.

Overall, the fertility rate of CAH patients has been greatly improved, from the common 21OHD to other rarer subtypes. This is a result of various factors, including the earlier diagnosis and better adherence to treatment; improved understanding of how estrogen, androgen, and progesterone affect ovulation and endometrium; and the wider application of ART. A cohort study showed that 14.7% of CAH women had children without ART and 2.4% with ART (20). There are several situations where ART use has prominent merits. The first and most common indication is anovulation, which may be secondary to high progesterone (as in 21OHD, 17OHD, and POR deficiency), high androgen (as in 11OHD), or low estrogen (as in StAR deficiency). These abnormalities disrupt the hypothalamus–pituitary–ovarian axis, leading to impaired follicular development and diminished LH surge. Some patients may return to regular ovulation after corticoid treatment, and ovulation induction is needed when ovulation fails to be restored. The second and most important indication is the detrimental effect of high progesterone on endometrium receptivity. When selecting the protocol for ovarian stimulation under high progesterone, the long GnRHa protocol was a popular choice in the hope of lowering progesterone, but the effect was somewhat limited. In fact, progesterone during the follicular phase did not affect oocyte quality and helped to prevent premature LH surges (77). With the strategy of frozen-embryo transfer, we could circumvent the adverse effect of progesterone on the endometrium. In endometrium preparation, the key lies in the suppression of endogenous progesterone to below 0.45 ng/ml by use of glucocorticoid (and mineralocorticoid when necessary) (72). The third indication is preimplantation genetic testing. Because of the autosomal recessive nature of CAH, genotyping the partner is recommended before pregnancy. If the husband was heterozygous for the same gene, preimplantation testing and thus ART were advisable (33).

The miscarriage rate of 18.2% was significantly elevated in CAH patients, as compared to 11.8% in the women receiving ART treatment (78) and 15.3% for the total population (79), but the reason remains unclear. About 48% of early pregnancy loss was due to chromosomal abnormalities, and advanced maternal age was an important determinant (80). In our study, the mean pregnancy age of all included studies was below 35, so advanced maternal age was not our prime suspect. No testing of the chorionic villi was ever reported in miscarriage cases of CAH patients, and it may be a direction for future research. We subgrouped the results of miscarriage rate by body mass index (BMI) (average BMI ≥25 or <25) and type of CAH (non-classical or assorted) and found no significant difference between subgroups. Given the currently limited data from retrospective studies, glucocorticoid treatment did not significantly affect the miscarriage rate of non-classical CAH patients. Another possible reason for the increased miscarriage rate is insufficient luteal support, which is not uncommon in CAH patients (81).

Unexpectedly, the elective abortion rate reached 5.5% among CAH patients, which was higher than the global rate of 3.9% (82). The elective abortion rate was significantly higher in those studies with a larger proportion of classic CAH than those with only non-classical patients, which indicated that the severity of the disease was the main cause of abortion. On the one hand, patients with classical CAH were usually under the impression of infertility, so birth control might be overlooked, which results in unintended pregnancies. On the other hand, women with severe CAH were more disadvantaged in education, employment, and marital status, which might explain the increased abortion rate (83).

Women with CAH are expected to be more vulnerable to gestational diabetes mellitus, because of the increased prevalence of obesity, insulin resistance, hyperglycemia, and corticoid treatments (9, 84). Our results showed that the risk of gestational diabetes was elevated [RR 2.67 (1.29–5.12)]. However, the absolute prevalence of GDM in CAH patients was 7.3%, which is comparable to 7.49% of singleton pregnancies of natural conception and 8.47% of singleton pregnancies after ART (85). The discrepancy of these results may lie in the small number of studies (n = 3) and CAH patients (n = 432) included in the study of relative risk. As for risk factors of GDM, the proportion of overweight and obesity raised our concern, as evidenced in **Table 2** that the average BMI in four studies reached or exceeded 25 kg/m^2^. However, among five studies that reported GDM prevalence, only two reported BMI, so further analyses of how BMI affects the GDM rate among CAH patients were not allowed. Since the age-adjusted risk of GDM increased with increasing BMI category among all ethnic groups (86), we recommended a better control of BMI before pregnancy for CAH patients. In addition, one study also proposed that keeping BMI below 23.36 kg/m would improve the pregnancy rate of embryo transfer among non-classical 21OHD women (72).

Twin pregnancies were important risk factors for all pregnancy complications. The rate of multiple gestations was high for rare types of CAH (as illustrated in **Table 1**) but was moderate for 21OHD patients (as shown in **Table 2**). We postulated that more follicles were stimulated or more embryos were transferred to increase the opportunity of pregnancy in rare types of CAH, the decision of which should be prudent to improve pregnancy outcome.

Alarmingly but not surprisingly, the rate of cesarean section nearly quadrupled in CAH women. This was mostly due to small maternal pelvis, vaginal stenosis, and fear of vaginal tear at parturition and sometimes due to severe hypertensive disorders during pregnancy. Interestingly, three single-center studies reported either 0% or 100% C-section rate among classical and non-classical 21OHD patients (65, 68, 70), which reflected, to some extent, how the tendency of the clinicians might affect the choice of mode of delivery.

There are several limitations to this study. First, the estimates of pregnancy complications were limited to 21OHD, since other subtypes were too rare. However, different subtypes and different mutations in the same gene could have distinct manifestations and thus various risks of pregnancy complications. Second, the number of studies included in the calculation of relative risk was limited, and the validity of the results was therefore impaired. Future studies are called for, especially cross-sectional census or multicenter studies. Third, the summary of the rare types of CAH is susceptible to selection bias, due to the fact that women with milder deficiencies are easier to get pregnant. Fourth, when applying these results to assess individual risks in the clinical setting, other factors needed to be taken into consideration, such as age, ethnicity and previous pregnancy history, which are not discussed in this research.

## 5 Conclusions

In our study, we summarized the clinical manifestations and considerations of ART use in rare types of CAH. As the diagnosis and treatment are improving, fertility issues should be fully addressed with all types of CAH patients. Women should be aware of their fertility possibilities and accessible fertility treatment. If they are reluctant to or not appropriate for pregnancy, information on contraception should be provided to decrease the elective abortion rate, especially for the more severe types of CAH patients. If women have fertility desire, fertility treatment could be designed according to their mutations and clinical manifestations. Multiple gestations should be avoided by reducing multiple follicular developments during ovulation induction or the number of embryos transferred. Better control of BMI may be beneficial to embryo implantation and the prevention of GDM. Glucocorticoid treatment didn’t have a significant effect on preventing miscarriage in non-classical CAH patients. Should miscarriage happen, a diagnostic workup is necessary. Overall, by establishing the prevalence and relative risk of pregnancy complications in CAH patients, we made the initial step toward prevention. Future studies are urgently needed to address whether different types of CAH affect the risk of pregnancy complications and to find out other interventions that are beneficial to pregnancy outcomes.

## Data availability statement

The original contributions presented in the study are included in the article/**Supplementary Material**. Further inquiries can be directed to the corresponding author.

## Author contributions

XG and JS designed the study. XG, YZ and YY performed literature searches, study selection, data extraction, and quality assessment. LZ, and KU did the data analyses. XG wrote the initial draft of the manuscript, MJ and BJ contributed to the writing of discussion. JS coordinated the study and make revisions of the manuscript. All authors contributed to the article and approved the submitted version.

## Funding

Research fund for Young Scholars of Hangzhou Medical College (KYQN202127); Adjunct Talent Fund of Zhejiang Provincial People’s Hospital.

## Conflict of interest

The authors declare that the research was conducted in the absence of any commercial or financial relationships that could be construed as a potential conflict of interest.

## Publisher’s note

All claims expressed in this article are solely those of the authors and do not necessarily represent those of their affiliated organizations, or those of the publisher, the editors and the reviewers. Any product that may be evaluated in this article, or claim that may be made by its manufacturer, is not guaranteed or endorsed by the publisher.

## Supplementary material

Appendix 1 Literature Search Strategy.

Appendix 2 PRISMA checklist.

Appendix 3 Quality Assessment of included studies.
