# Erratum: Getting pregnant with congenital adrenal hyperplasia: assisted reproduction and pregnancy complications. A systematic review and meta-analysis

**DOI:** 10.3389/fendo.2023.1276185

**Published:** 2023-09-28

**Authors:** Frontiers Production Office

**Affiliations:** Frontiers Media SA, Lausanne, Switzerland

**Keywords:** congenital adrenal hyperplasia (CAH), assisted reproduction technology (ART), pregnancy complication, meta-analysis, systematic review, miscarriage, abortion (induced), glucocorticoid therapy

Due to a production error, there was a mistake in **Figure 1** in the article PDF as published. **Figure 1** was a duplicate of Figure 2. The corrected **Figure 1** appears below.

**Figure 1:**
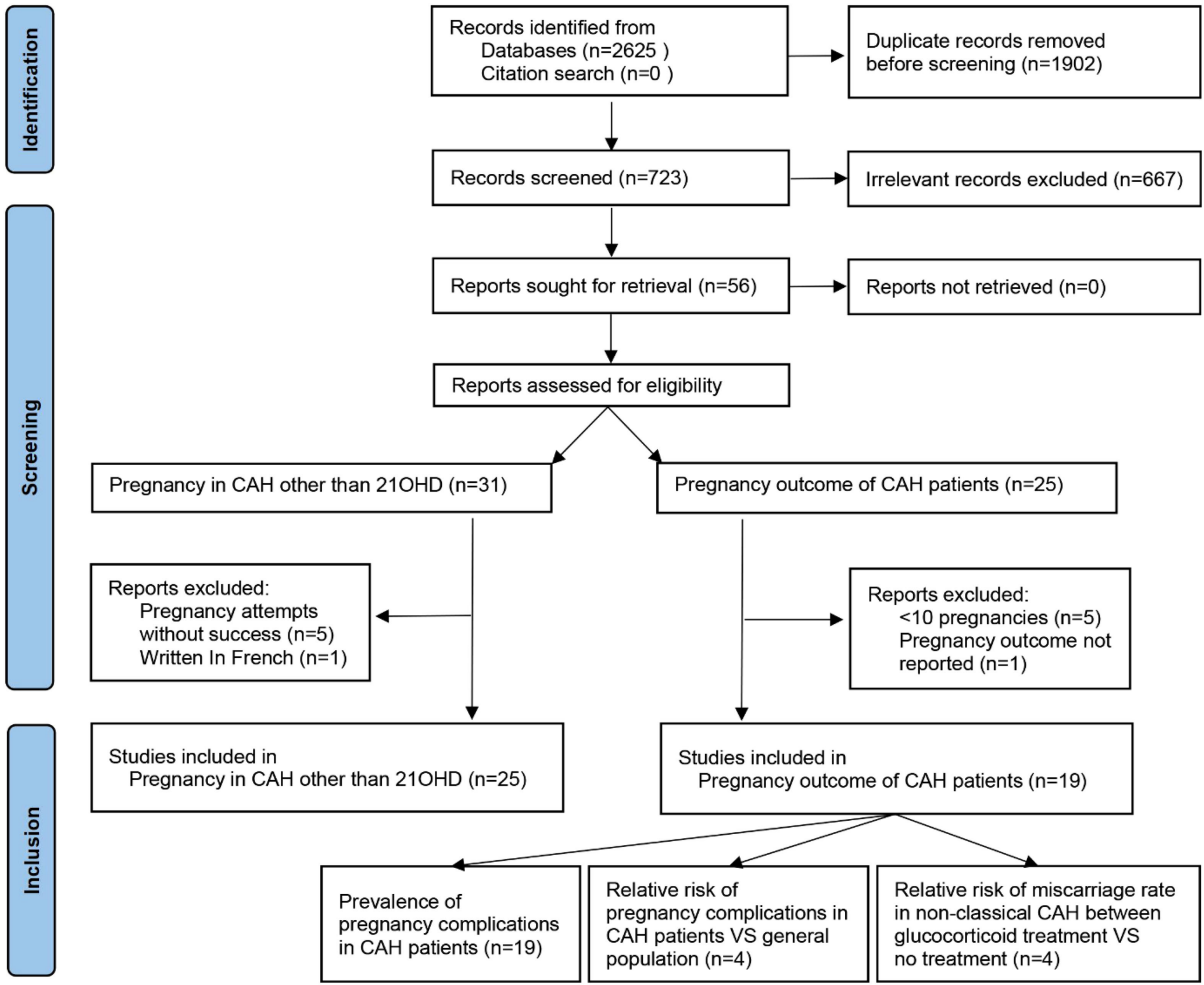
PRISMA flowchart of literature search and selection. PRISMA, Preferred Reporting Items for Systematic Reviews and Meta-Analyses.

The publisher apologizes for this mistake. The original version of this article has been updated.

