# Corrigendum: Getting pregnant with congenital adrenal hyperplasia: assisted reproduction and pregnancy complications. A systematic review and meta-analysis

**DOI:** 10.3389/fendo.2023.1269711

**Published:** 2023-09-28

**Authors:** Xiaoyan Guo, Yu Zhang, Yiqi Yu, Ling Zhang, Kamran Ullah, Mengxia Ji, Bihui Jin, Jing Shu

**Affiliations:** ^1^ Center for Reproductive Medicine, Department of Reproductive Endocrinology, Zhejiang Provincial People’s Hospital, Affiliated People’s Hospital, Hangzhou Medical College, Hangzhou, China; ^2^ School of Nursing, Hangzhou Medical College, Hangzhou, China; ^3^ Department of Biology, The University of Haripur, Haripur, Pakistan

**Keywords:** congenital adrenal hyperplasia (CAH), assisted reproduction technology (ART), pregnancy complication, meta-analysis, systematic review, miscarriage, abortion (induced), glucocorticoid therapy

In the published article, there were two errors in **Figure 5** as published. “Odds ratio” was mistakenly used instead of “relative risk” in the code to generate **Figure 5**. Furthermore, the data from two studies were incorrectly cited (Eyal, et al., 2017 and Moran, et al., 2006). The corrected **Figure 5** and its caption appear below.

Consequently, in the published article there were 5 errors related to the description of **Figure 5**. A correction has been made to **Abstract**. This sentence previously stated:

“Glucocorticoid treatment in non-classical CAH patients significantly lowered the miscarriage rate when compared to the untreated group (RR 0.25 (0.13-0.47)).”

The corrected sentence appears below:

“The miscarriage rate in non-classical CAH patients was not significantly different with or without glucocorticoid treatment from retrospective studies.”

A correction has been made to **2 Methods**, *2.3 Data analysis*. This sentence previously stated:

“When a control group without adrenal insufficiency was provided, a crude odds ratio (OR) and 95% CI were calculated using the Mantel-Haenszel method based on a random-effects model.”

The corrected sentence appears below:

“To measure dichotomous outcomes, a relative risk (RR) and 95% confidence interval (CI) were calculated using the Mantel-Haenszel method based on a random-effects model.”

A correction has been made to **3 Results** *, 3.2 Pregnancy complications of congenital adrenal hyperplasia patients*, paragraph one.

This sentence previously stated:

“The miscarriage rate of the glucocorticoid treatment group is significantly lowered than that of the untreated group in the non-classical type of CAH patients (RR 0.25 (0.13–0.47)), as shown in **Figure 5**.”

The corrected sentence appears below:

“The risk of miscarriage in non-classical CAH patients was not significantly influenced by glucocorticoid treatment, as shown in **Figure 5**.”

A correction has been made to **4 Discussion**, paragraph three.

This sentence previously stated: “However, glucocorticoid treatment in the non-classical type of CAH patients significantly lowered the miscarriage rate (RR 0.25 (0.13–0.47)). Therefore, proper glucocorticoid treatment might be the key.”

The corrected sentence appears below:

“Given the currently limited data from retrospective studies, glucocorticoid treatment did not significantly affect the miscarriage rate of non-classical CAH patients”.

A correction has been made to **5 Conclusions**.

This sentence previously stated:

“For the non-classical type of CAH, glucocorticoid treatment is recommended to prevent miscarriage.”

The corrected sentence appears below:

“Glucocorticoid treatment didn’t have a significant effect on preventing miscarriage in non-classical CAH patients.”

The authors apologize for these errors. The original article has been updated.

## Figures and Tables

**Figure 5:**
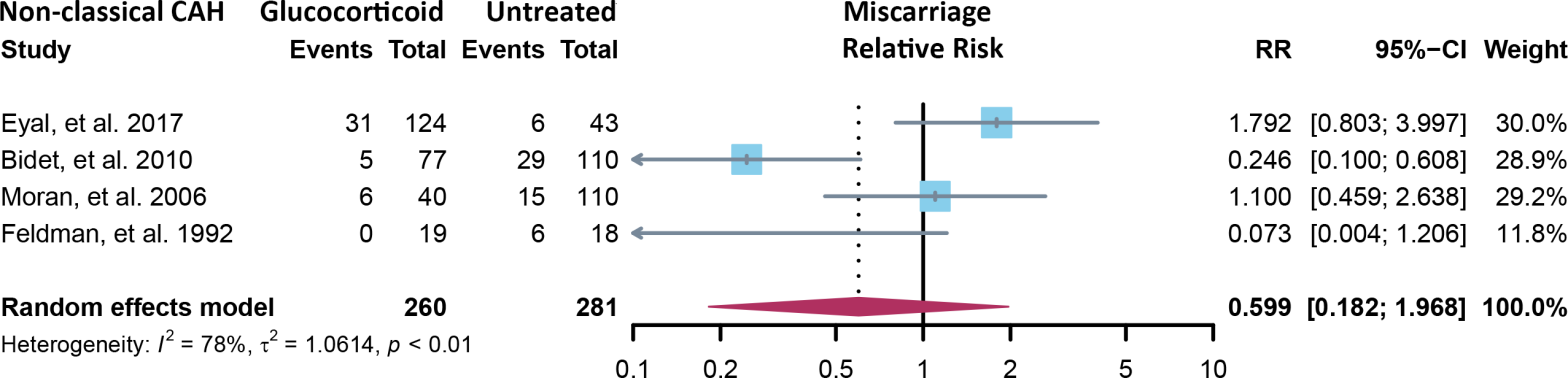
Relative risk of miscarriage in the non-classical form of CAH between glucocorticoid treatment and non-treatment. CAH, congenital adrenal hyperplasia.

