# Supplementary material for: Getting pregnant with congenital adrenal hyperplasia: Assisted reproduction and pregnancy complications. A systematic review and meta-analysis
Source: Front Endocrinol (Lausanne). 2022 Aug 31;13:982953. doi: 10.3389/fendo.2022.982953 (PMC9470834; doi:10.3389/fendo.2022.982953)
Supplement: Appendix 1 — Literature Search Strategy. [file DataSheet_1.docx]

Search strategy in Pubmed

Search: ("Congenital Adrenal Hyperplasia"[Title/Abstract] OR "21-hydroxylase"[Title/Abstract] OR "P450c21"[Title/Abstract] OR "CYP21A2"[Title/Abstract] OR "steroidogenic acute regulatory protein"[Title/Abstract] OR "17alpha-hydroxylase"[Title/Abstract] OR "17,20-lyase"[Title/Abstract] OR "CYP17A1"[Title/Abstract] OR "P450c17"[Title/Abstract] OR "P450 cholesterol side-chain cleavage"[Title/Abstract] OR "P450scc"[Title/Abstract] OR "CYP11A1"[Title/Abstract] OR "P450 oxidoreductase"[Title/Abstract] OR "CYB5A"[Title/Abstract] OR "cytochrome b5"[Title/Abstract] OR "11beta-hydroxylase"[Title/Abstract] OR "CYP11B1"[Title/Abstract] OR "aldosterone synthase"[Title/Abstract] OR "CYP11B2"[Title/Abstract] OR "HSD3B2"[Title/Abstract] OR "3β-hydroxysteroid-dehydrogenase"[Title/Abstract])

AND

("pregnan*"[Title] OR "outcome"[Title] OR "birth"[Title] OR "fertili*"[Title] OR "fecundity"[Title] OR "reproducti*"[Title] OR "deliver*"[Title] OR "gestation*"[Title])
